# Patient with post infective demyelinating disease for dynamic hip screw repair under epidural anaesthesia

**DOI:** 10.4103/0019-5049.79884

**Published:** 2011

**Authors:** Pritee H Bhirud, Dhanwanti Rajwade, Ragini Suchak

**Affiliations:** Bhabha Atomic Research Centre Hospital, Anushakti Nagar, Mumbai 400094, India

Sir,

We report a case of a 47-year-old female weighing 35 kg, height - 150 cm, of Post Infective Chronic Demyelinating Disease, for a Dynamic Hip Screw of right intertrochanteric fracture femur. She was diagnosed with chronic demyelinating disease 3 years ago following Falciparum malaria. Magnetic resonance imaging showed tiny nodular, hyperintense lesion in right corpus callosum, possibly lacunar infarct/focal demyelination. After injection artesunate, dopamine infusion for 48 hours, injection Methylprednisolone 500 mg BD, she improved, with residual lower limb weakness, ptosis and dysarthria. She walked minimally until 2 weeks ago when she sustained a right-sided intertrochanteric fracture, following a fall. She was conscious, oriented, speech suggestive of spastic dysarthria, right eye ptosis with bilateral restricted extraocular movements. Other cranial nerves were normal.

**Figure 1:**
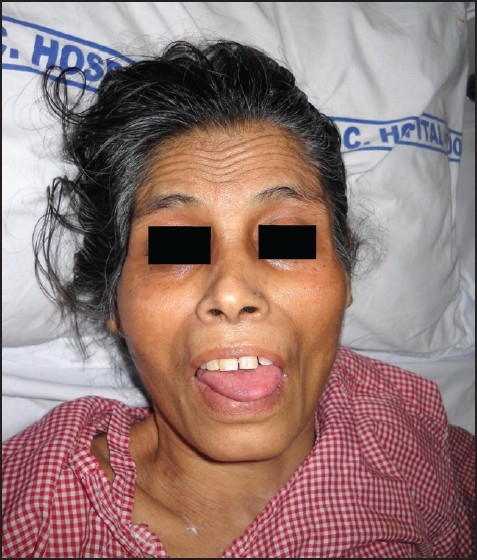
Anterior view showing restricted mouth opening

All limbs had hyperreflexia, spasticity, muscle wasting, motor power 3/5, equivocal Babinski’s sign and ill-sustained clonus. Normal motor coordination, no involuntary movements, negative cerebellar signs and normal sensory system were observed. *The Autonomic Nervous system* tests evaluating variations in pulse related to position, sustained hand grip and blood pressure variations with position, deep breathing and Valsalva’s maneuver were *abnormal*. Bladder and bowel movements were normal. *Airway Examination* showed limited mouth opening of 1.5 cm [Figure 1], exaggerated jaw reflex and temporomandibular joint spasticity.

Repeat MRI was stable. Hb,10.5; Serum K, 4.0; routine investigations were normal.

American Society of Anaesthesiologists’ (ASA)-Physical Status III consent was taken in view of difficult airway. Injection hydrocortisone 100 mg IV was given. Difficult Airway Cart, warming blanket and emergency drugs were kept at standby. We instituted epidural block with 16 G catheter in situ through Tuohy needle under absolute aseptic precautions in the 3-4 lumbar space, using negative pressure to saline technique, in left lateral position. Test dose: 2 ml 2% lignocaine + 8 ml 0.5% bupivacaine after 5 minutes was given in supine position, + 2 ml increment after 10 minutes. There was a significant fall in blood pressure, unaccompanied by tachycardia [Table 1]. Hypotension was corrected by injection ephedrine 6 mg bolus, total of 30 mg. A level of T12 was obtained and surgery was conducted without intraoperative complications. Epidural top up was given after 1.5 hours with 5 ml 2% lignocaine. Post-operatively, she was monitored for neurological deficits and was found to be stable. On demand, post-operative analgesia was provided through epidural catheter with injection tramadol 50 mg in 8 ml normal saline for 48 hours.

**Table 1:** Intraoperative haemodynamic values

	Time	P	SBP (mmHg)	DBP (mmHg)	MBP (mmHg)	Ephedrine iv bolus
Baseline	12:16	80	132	79	99	
after epidural	12:25	82	115	85	98	
	12:35	82	92	63	76
	12:39	88	101	71	51
	12:41	92	91	60	72	
	12:44	90	82	52	64	6 mg
	12:45	87	79	54	65	6 mg
	12:46	88	90	62	73	6 mg
	12:48	81	101	73	85	
Epidural top up	12:56	87	94	63	77	
	13:01	84	81	51	60	6 mg
	13:05	76	101	60	75	
Colloid	13:11	78	100	64	76	
	13:16	81	97	61	77	
	13:21	80	96	60	76	6 mg
	13:26	75	99	64	79	
0.5 mg midazolam iv	13:31	74	104	63	76	
	13:36	80	95	64	78	
	13:40	86	98	60	76	

P: Pulse; SBP: Systolic blood pressure; DBP: Diastolic blood pressure; MBP: Mean blood pressure

Prior to conducting the case, we researched post-infective demyelinating diseases. Some patients experienced a neurological syndrome after *complete recovery from Plasmodium falciparum* infection, which is named as Post Malaria Neurological Syndrome, characterised by acute onset confusion, epileptic seizures/neurological/ psychiatric signs within days to weeks.[1]

Our patient had neurological involvement during malaria and sustained residual neurological deficits. Since these are rare occurrences, we drew knowledge for conducting this case based on reports on Multiple Sclerosis (MS). General anaesthesia was not preferred since succinylcholine, our natural choice in view of difficult airway, is contraindicated in chronic demyelinating diseases,[2] as increased muscle membrane sensitivity causes excessive potassium release. The blood-brain barrier defect, pre-existing atrophic muscles and impaired neuromuscular transmission caution use of non-depolarising muscle relaxants as well. Coexisting autonomic dysfunction impairs compensatory responses to hypotension of induction agents.[2] Demyelination produces conduction blockade, and lack of protective nerve sheaths around the spinal cord increase susceptibility to neurotoxicity of local anaesthetics.[2] Dripps and Vandam avoided spinal anaesthesia in patients with pre-existing central nervous system disorders like MS, amyotrophic lateral sclerosis and post-polio syndrome.[3] However, Hebl *et al*.[4] reported no exacerbation of post-operative neurodeficits in patients with pre-existing central nervous system disorders given centrineuraxial blocks. The epidural analgesia did not increase progressive neurological deficits in parturients with MS.[5] As local anaesthetic concentration is significantly smaller within spinal cord white matter after epidural administration,[2] and causes graded hypotension, we preferred this modality over intrathecal block. Given risks of general anaesthesia in patients with demyelinating disease, surgical site and multiple studies reporting regional techniques, we can successfully conduct such cases under epidural block.
